# Repeatability and comparability of the Galilei-G4 and Cassini in measuring corneal power and astigmatism in normal and post-refractive surgery eyes

**DOI:** 10.1038/s41598-021-94319-w

**Published:** 2021-08-09

**Authors:** Mohamed Mohamed, Mahmood Khan, Amritha Kanakamedala, Isha Gupta, Li Wang, Douglas Koch, Zaina Al-Mohtaseb

**Affiliations:** 1 Department of Ophthalmology, Cullen Eye Institute, Baylor College of Medicine, 6565 Fannin, NC205, Houston, TX 77030 USA; 2 Department of Ophthalmology, Weill Cornell Ophthalmology, Weill Cornell, New York, NY USA

**Keywords:** Refractive errors, Eye diseases, Eye abnormalities

## Abstract

To assess the repeatability and comparability of the Galilei G4 versus the Cassini topographer in post-refractive eyes and in normal eyes, including older patients representative of an initial cataract evaluation. Simulated keratometric (simK), total corneal and posterior corneal power and astigmatism were evaluated in both post-refractive and normal eyes. Repeatability was measured by calculating within-subject standard deviation (Sw), coefficient of variation (CoV), and intraclass correlation coefficient (ICC). Vector analyses and Bland–Altman plots were employed to assess agreement between devices. We studied 32 subjects with a history of refractive surgery and 32 subjects without a history of refractive surgery undergoing cataract surgery. The mean age was 55 ± 18.5 years and the age range was 21.5–91.5 years. In non-refractive and post-refractive eyes, the ICC was > 0.9 (P < 0.001) for all corneal powers and for simK and total corneal astigmatism for both analyzers. The ICC for posterior corneal astigmatism magnitude using the Galilei was 0.62 and 0.67 and for the Cassini 0.55 and 0.38 in normal and post-refractive eyes, respectively. In both post-refractive and normal eyes, the Galilei G4 and Cassini analyzers have high repeatability in simK, total, and posterior corneal power and low repeatability for posterior corneal astigmatism.

## Introduction

Accurate measurements of corneal curvature, pachymetry, and power are necessary for successful outcomes in refractive procedures and cataract removal surgery^1–3^. Corneal topography is important in the pre-operative assessment for patients undergoing photorefractive keratotomy (PRK) or laser in situ keratomileusis (LASIK). Similarly, topography is a key factor for screening of toric and multifocal lens implants as well as confirming biometry prior to replacing the natural lens in cataract surgery. There are a number of different devices that can measure astigmatism and corneal power, such as manual and automatic keratometers, Placido-based corneal topographers, scanning slit corneal topographers, low-coherence reflectometers, swept source OCT biometer such as IOLMaster 700, and Scheimpflug image-based topographers such as Galilei G4 Dual Scheimpflug Analyzer^4,5^.

The Cassini is the first commercially available topographer that utilizes point-source color light emitting diode (LED) technology. The device creates a reflection of 679 LEDs to measure anterior corneal curvature and is able to evaluate corneal posterior surface via 2nd Purkinje Imaging Technology^6^. The Galilei G4 Dual Scheimpflug Analyzer is another device used to measure corneal topography. This device utilizes Scheimpflug imaging and a Placido disk to create a three-dimensional measurement of the anterior segment^7,8^. Devices that utilize both Placido disk and Scheimpflug imaging can measure beyond the anterior surface and can assess the corneal thickness and posterior curvature.

Given the availability of several devices, there currently lacks a gold standard in measuring corneal power and astigmatism as a means for pre-operative evaluation. One method of determining the clinical accuracy of these devices is by comparing the repeatability and reproducibility of these instruments^9^. Repeatability is the variability of results measured in short intervals, while reproducibility is defined as the variability of results measured under different circumstances (e.g. multiple operators). Comparability is based on comparing data between different devices and accuracy defined as the closeness of a measured value to a standard value. Authors from the Cullen Eye Institute at Baylor College of Medicine have previously assessed the repeatability and comparability of anterior corneal power and astigmatism measurements of the color LED topographer (Cassini), Placido-based cornea topographer, and low coherence reflectometer^5^. To date, there has been only one study evaluating the repeatability and comparability of corneal power and astigmatism of the Cassini and Galilei G4 Dual Analyzer in normal and post-refractive eyes; however, this was done in young volunteers, aged 18–40 years^10^ not in the typical cataract population.

Among US patients, the median age at initial cataract surgery has been shown to be around 60 years in some communities and nearly 80 years in others^11^. Our study evaluated corneal power and astigmatism parameters to determine which measurements of the Cassini and Galilei G4 analyzers are repeatable and comparable incorporating older patients representing the typical age bracket of cataract patients.

## Patients and methods

This study was performed in accordance with the ethical standards established in the 1964 Declaration of Helsinki. We received Institutional Review Board (IRB) approval from Baylor College of Medicine for a prospective observational trial of corneal power and astigmatism in normal and post-refractive surgery eyes. All eyes were measured by a single observer (MK), and were performed at the Alkek Eye Center at Baylor College of Medicine. We confirm that all research was performed in accordance with relevant regulations and informed consent was obtained from all participants.

### Patients

This study enrolled two groups of subjects: (1) 32 patients (32 eyes) with no history of refractive surgery and (2) 32 patients (32 eyes) who had previously undergone myopic LASIK or PRK. At least 32 eyes were required to produce a significance level of 5% and a test power of 80%. This was computed by a sample size calculation to detect a difference of one-half of the standard deviation of differences in values between the two devices.

The minimum interval between refractive surgery and measurement was 3 months for LASIK and 6 months for PRK. All patients were informed of the nature of the study, and written consent was obtained to have additional exams in addition to the regular screen or follow-up exams. Subjects were screened for anterior and posterior segment disease and excluded if they had a history of previous ocular surgery, trauma, corneal or ocular pathology including dry eye syndrome, and contact lens wear within 2 weeks of measurement time. The criteria for dry eye exclusion included patients with known previous diagnosis of Sjögren syndrome or Dry Eye Syndrome. In addition, patients with signs of punctate epithelial erosions seen on slit lamp or noted on corneal topography were excluded from the study.

### Measurements

All measurements obtained for anterior, total, and posterior corneal power and astigmatism measurements were performed with the Galilei G4 (Ziemer, Zürich, Switzerland software version v6.4.1) and original Cassini (i-Optics, The Hague, Netherlands software version v2.5.0) corneal imaging systems. Three consecutive measurements were obtained in one randomly selected eye of each subject to assess intra-observer repeatability. Due to changes in the tear film that can affect Placido disc measurements and quality of corneal images, the operator waited 4 s between the blink and the capture of each image. Following each measurement, the subjects were allowed to sit back for 20 s to allow for device re-alignment and to allow for formation of an adequate tear film. Subjects were instructed to blink before each measurement.

We included only scans that met the manufacturer’s minimally acceptable quality factors. For the Galilei G4, the internal software calculates the percentages for the quality parameters, which include motion compensation, placido, Scheimpflug, and motion distance; the minimum values were 85%, 85%, 90%, and 70%, respectively^12^. Similarly, the Cassini’s quality factors were centration, focus, corneal coverage, and stability; the minimum values were 85% for all parameters.

The parameters measured were mean simulated keratometry power (simK), total corneal power, and posterior corneal power. For each of the corneal powers, corneal astigmatism (D) was recorded as the dioptric value given by each device. In the Cassini and Galilei G4, a keratometric index of 1.3375 was used to calculate the powers of the steep and flat meridians as used in previous studies^12^.

### Statistical analysis

Statistical analysis was performed using R software (version 0.99.903, R Foundation for Statistical Computing, Vienna, Austria)^13^. Intra-observer repeatability was assessed by calculating the within-subject standard deviation (S_W_), coefficient of variation (CoV), and intra-class correlation coefficient (ICC), as described by Kim et al.^12^. The S_W_ is defined as the standard deviation of repeated measurements^14^. The CoV represents the ratio of the S_W_ to the mean, with a lower CoV signifying higher repeatability. The ICC is a measure of consistency between repeated measurements; it ranges from 0 to 1, with 1 indicating complete agreement; we used a scale of ≥ 0.90 = high agreement, 0.75–0.90 = moderate agreement, and < 0.75 = low agreement; as classified by Portney and Watkins^15^.

Bland–Altman analysis was performed to evaluate the differences between repeated measurements of corneal power and astigmatism. The statistical analysis did not depend on whether the anterior or posterior cornea was chief WTR and ATR. The 95% limits of agreement (LoA) were calculated as the mean difference ± 1.96 SD. For sample size determination, with three repeated measurements per subject and confidence interval of 20% on either side of the estimate of S_w_, the calculated sample size was 30 subjects. Data distribution for normality was checked using the Kolmogorov–Smirnov test. Comparison of mean values between devices was assessed using either paired student t test or Wilcoxon test depending on the data distribution.

## Results

Thirty-two eyes (15 right eyes, 17 left eyes) of 32 subjects (16 male, 16 female) with no history of refractive surgery (normal eyes) and thirty-two eyes (15 right eyes, 17 left eyes) of 32 subjects (10 male, 22 female) with a history of myopic LASIK or PRK (post-refractive eyes) were studied. The mean ages of normal and post-refractive groups was 59.0 ± 18.8 (range 21–91 years) and 49.3 ± 16.4 years (range 26–77 years), respectively.

In Table 1 for normal eyes and Table 2 for post-refractive eyes, the mean value, S_W_, CoV, and ICC for all corneal power and astigmatism parameters are shown.

**Table 1:** Comparability and intra-observer repeatability for corneal power and astigmatism obtained from the Cassini and Galilei G4 in normal eyes.

	Mean ± SD (range) (D)	*P*-value*	Sw(D)	CoV(%)	ICC
**Galilei**					
Keratometric (SimK)	44.03 ± 1.65 (40.83–48.49)	–	0.07	0.14	0.998
Astigmatism	0.95 ± 0.76 (0.08–4.15)	–	0.15	19.10	0.956
Total cornea	42.71 ± 1.74 (39.35–47.34)	–	0.11	0.20	0.996
Astigmatism	0.91 ± 0.78 (0.22–4.35)	–	0.16	19.59	0.959
Posterior cornea	− 6.24 ± 0.28 (− 7.05–[− 5.87])	–	0.04	0.51	0.981
Astigmatism	− 0.31 ± 0.12 (− 0.55–[− 0.06])	–	0.09	39.15	0.616
**Cassini**					
Keratometric (SimK)	44.02 ± 1.62 (40.79–48.72)	0.63	0.17	0.29	0.988
Astigmatism	0.95 ± 0.78 (0.15–4.09)	0.78	0.12	16.68	0.959
Total cornea	42.91 ± 1.56 (39.76–47.41)	**0.004**	0.16	0.29	0.989
Astigmatism	0.92 ± 0.73 (0.23–3.99)	0.99	0.19	25.38	0.934
Posterior cornea	− 6.22 ± 0.26 (− 6.74–[5.76])	0.94	0.07	0.85	0.936
Astigmatism	− 0.34 ± 0.17 (− 0.72–[− 0.09])	0.33	0.12	32.22	0.554

Comparisons of corneal power and astigmatism obtained from the Cassini and Galilei G4 in normal eyes. simK = simulated keratometric; Sw = within-subject standard deviation, CoV = coefficient of variation, and ICC = intra-class correlation coefficient. Signifcant values with *P* < 0.05 are in bold. Values are expressed as mean ± SD.

*P values comparing mean values between Cassini and Galilei, and bold value indicating *P* < 0.05.

**Table 2:** Comparability and intra-observer repeatability for corneal power and astigmatism obtained from the Cassini and Galilei G4 in post-refractive eyes.

	Mean ± SD (range) (D)	*P*-value*	Sw(D)	CoV(%)	ICC
**Galilei**					
Keratometric (SimK)	40.97 ± 2.44 (36.40–46.75)	–	0.22	0.28	0.997
Astigmatism	0.71 ± 0.41 (0.13–2.07)	–	0.24	25.34	0.950
Total cornea	38.92 ± 2.61 (33.81–45.49)	–	0.21	0.30	0.997
Astigmatism	0.67 ± 0.42 (0.17–1.96)	–	0.15	23.97	0.909
Posterior cornea	− 6.35 ± 0.30 (− 6.90–[− 5.74])	–	0.03	0.45	0.999
Astigmatism	− 0.31 ± 0.16 (− 0.73–[− 0.09])	–	0.11	22.25	0.669
**Cassini**					
Keratometric (SimK)	40.81 ± 2.37 (36.40–46.28)	0.40	0.22	0.35	0.982
Astigmatism	0.95 ± 0.78 (0.15–4.09)	0.06	0.22	20.38	0.929
Total cornea	39.79 ± 2.31 (35.48–45.10)	0.08	0.24	0.39	0.988
Astigmatism	0.83 ± 0.45 (0.19–1.69)	0.11	0.27	28.00	0.904
Posterior cornea	− 6.02 ± 0.38 (− 6.59–[5.26])	**0.0001**	0.09	1.19	0.966
Astigmatism	− 0.34 ± 0.17 (− 0.72–[− 0.09])	0.08	0.14	30.98	0.380

Comparisons of corneal power and astigmatism obtained from the Cassini and Galilei G4 in post-refractive eyes. simK = simulated keratometric; Sw = within-subject standard deviation, CoV = coefficient of variation, and ICC = intra-class correlation coefficient. Signifcant values with *P* < 0.05 are in bold. Values are expressed as mean ± SD.

*P values comparing mean values between Cassini and Galilei, and bold value indicating *P* < 0.05.

In normal, non-refractive, eyes, the CoV was ≤ 0.51% for all corneal power measurements (simK, total, and posterior) and the S_W_ was ≤ 0.11 D with the Galilei; for all corneal astigmatism data with the Galilei, the CoV was ≤ 39.15% and the S_W_ was ≤ 0.16 D with the Galilei. Although the ICC for posterior corneal astigmatism was 0.616, the ICCs for all other corneal power and astigmatism magnitudes were > 0.9.

For the all corneal power Cassini measurements in normal eyes, the CoV was ≤ 0.85% and the Sw was ≤ 0.17 D; for all corneal astigmatism measurements with the Cassini, the CoV was ≤ 32.22% and the Sw was ≤ 0.19 D. The ICCs were > 0.9 for all corneal power and astigmatism magnitudes, with the exception of posterior corneal astigmatism, which was 0.554.

In post refractive eyes, the Sw was ≤ 0.22 D and the CoV was ≤ 0.45% for all corneal power measurements with the Galilei; the Sw was ≤ 0.24 D and the CoV was ≤ 25.34% for all corneal astigmatism measurements with the Galilei. The ICCs were > 0.9 for all corneal power and astigmatism magnitudes, except for posterior corneal astigmatism, which was 0.669.

For all corneal power measurements with the Cassini in post-refractive eyes with the Cassini, the Sw was ≤ 0.24. The CoV was 0.35%, 0.39%, 1.19% for simK, total, and posterior corneal power measurements. For all corneal astigmatism measurements with the Cassini, the Sw was ≤ 0.27 D and the CoV was ≤ 30.98%. Although the ICC for posterior corneal astigmatism which was 0.380, the ICCs were > 0.9 for all other corneal power and astigmatism magnitudes.

There was a statistically significant difference between the means from the Cassini and Galilei G4 devices (p < 0.01) for total corneal power in normal eyes and for posterior corneal power in post-refractive eyes.

**Figure 1:**
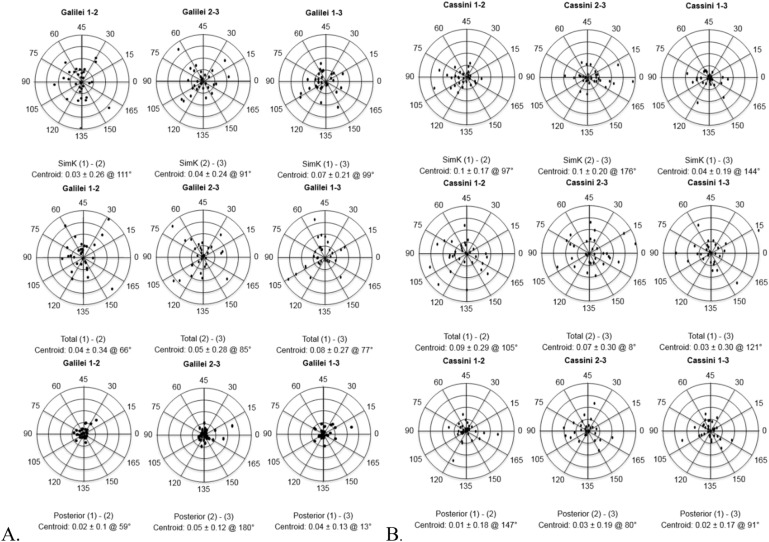
(**A**) Galilei and (**B**) Cassini, for normal eyes, double-angle plots showing intrasubject differences in simulated keratometric (SimK) (top row), total (middle row), and posterior corneal astigmatism (bottom row) measurements. Each ring represents 0.25 diopters (D), and the outer ring represents 1.0 D.

In normal eyes, the intrasubject differences in astigmatism between repeated measurements were within 0.75 D except for one simK plot under Galilei analysis, two of the simK plots under Cassini analysis as well as all posterior corneal astigmatism double angle plots in both the Cassini and Galilei (Fig. 1)^16^. Most total corneal astigmatism measurements in normal eyes were within 1.0 D, except for one plot under Cassini analysis with outliers > 1.0 D.

**Figure 2:**
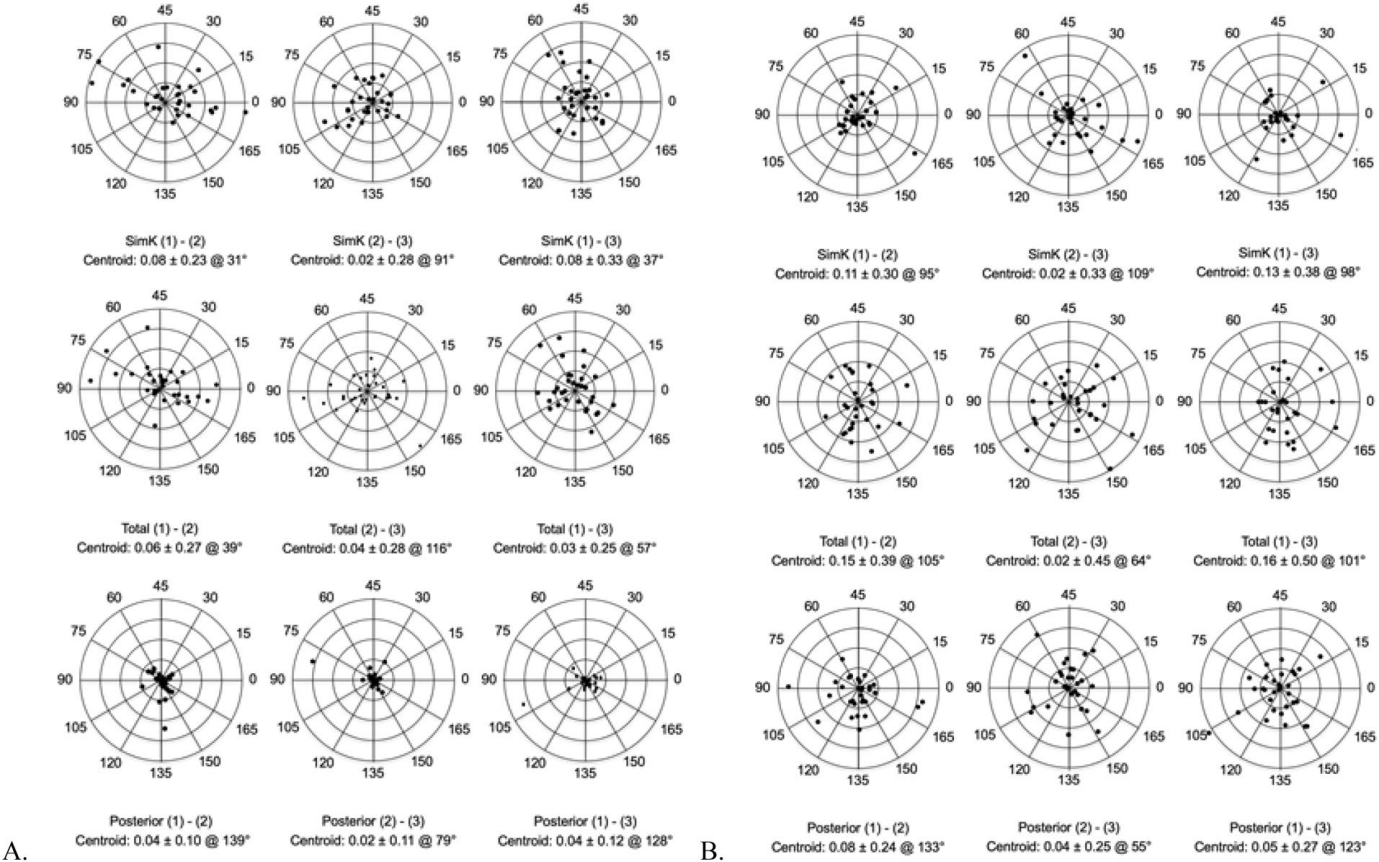
(**A**) Galilei and (**B**) Cassini, For post-refractive eyes, double-angle plots showing intrasubject differences in simulated keratometric (SimK) (top row), total (middle row), and posterior corneal astigmatism (bottom row) measurements. Each ring represents 0.25 diopters (D), and the outer ring represents 1.0 D.

In post-refractive surgery eyes, the differences in astigmatism magnitude were within 1.0 D for all measurements, except for one plot measuring simK astigmatism under Galilei analysis and one pair of plots measuring total corneal astigmatism each in the Cassini and Galilei groups with outliers > 1.0 D (Fig. 2)^16^.

**Figure 3:**
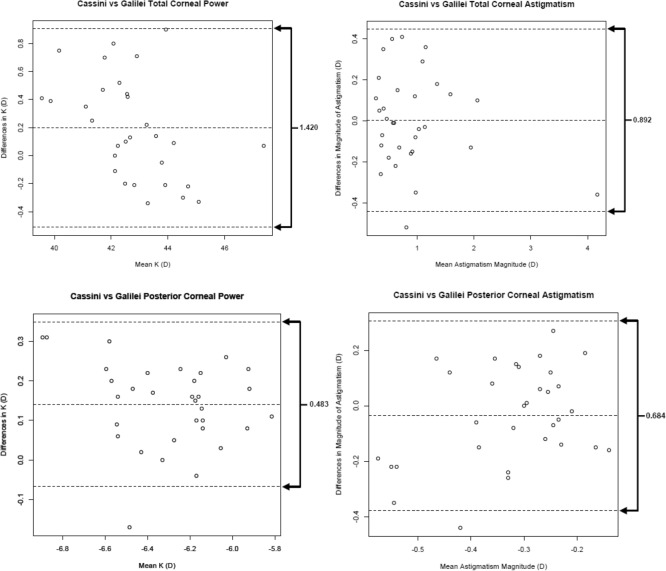
Bland–Altman plots showing differences in corneal power and astigmatism for repeated measurements in normal eyes. The differences were taken between the first and second measurements for each subject. The mean difference is represented by the solid line, and 95% limits of agreement (LoA) are represented by dotted lines. (Top left) total corneal power, (top right) total corneal astigmatism, (bottom left) posterior corneal power, and (bottom right) posterior corneal astigmatism.

**Figure 4:**
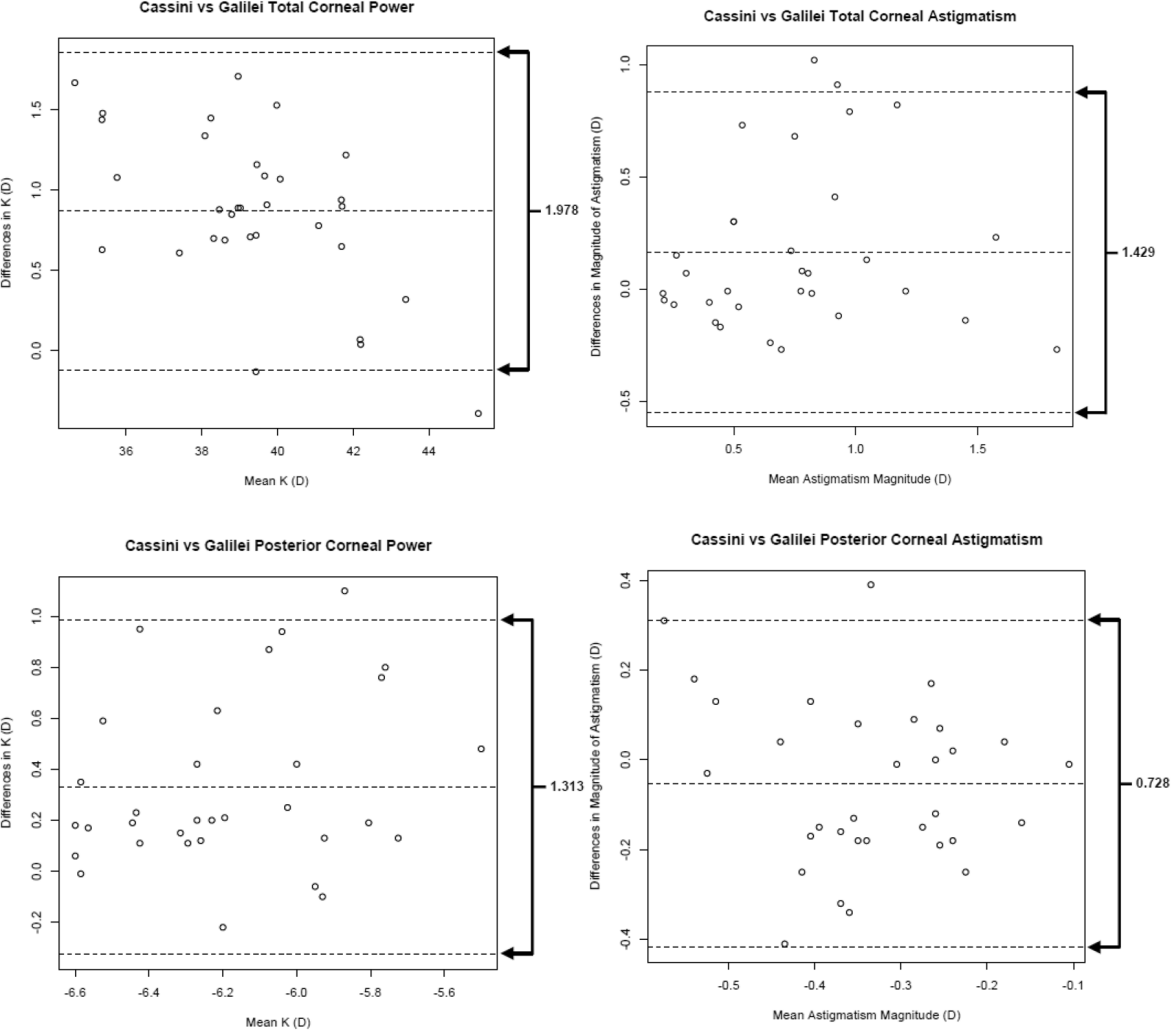
Bland–Altman plots showing differences in corneal power and astigmatism for repeated measurements in post-refractive surgery eyes. The differences were taken between the first and second measurements for each subject. The mean difference is represented by the solid line, and 95% limits of agreement (LoA) are represented by dotted lines. (Top left) total corneal power, (top right) total corneal astigmatism, (bottom left) posterior corneal power, and (bottom right) posterior corneal astigmatism.

In post-refractive eyes, the 95% LoA between repeated measurements for posterior and total corneal power and astigmatism were larger than in normal eyes. In normal eyes, the 95% LoA were smaller in posterior corneal astigmatism and power than for total corneal astigmatism and power, as well as in post-refractive eyes (Figs. 3 and 4)^16^. In both normal and post-refractive eyes, outliers beyond the 95% LOA were seen in all corneal astigmatism and power measurements.

## Discussion

The purpose of this study was to assess the repeatability and comparability of the dual Scheimpflug–Placido topographer (Galilei G4 Dual) versus the light-emitting diode topographer (Cassini) in normal and post-refractive surgery eyes including subjects with a typical age of cataract patients. The mean age of our cohort was 55 ± 18.5 years. Both the Galilei G4 Dual and Cassini showed high intra-observer repeatability for all corneal power measurements in normal and post-refractive eyes. With regards to corneal astigmatism, both the Galilei G4 Dual and Cassini showed high intra-observer repeatability in normal and post-refractive eyes for simulated keratometric (simK) and total corneal astigmatism. Posterior corneal astigmatism, in normal and post-refractive eyes, however, showed low intra-observer repeatability using both the Galilei G4 Dual and Cassini.

Wang et al. found that the dual Scheimpflug analyzer has high repeatability in measurements of corneal power, anterior chamber depth (ACD), corneal aberrations, and pachymetry^17^. Cervino found moderate repeatability for wavefront aberrations and astigmatism and high repeatability for corneal power and central pachymetry^18^. Kim et al. furthermore assessed the repeatability of the Scheimpflug analyzer in simK, total and posterior corneal curvature measurements in both normal and post-refractive surgery eyes and found high repeatability in all measurements, except for posterior corneal astigmatism which was moderately repeatable^12^. Lu et al. found high repeatability in both normal and post-refractive eyes for Cassini and Sirius (Placido-Scheimpflug) systems for simK, total corneal power and astigmatism recordings except for low repeatability in total corneal astigmatism in post-refractive eyes^19^. In comparing the ICC between Cassini and Galilei, Lee et al. found high repeatability for simK power measurements in normal (0.991) and post-refractive surgery eyes (0.986), and poor repeatability in posterior corneal astigmatism measurements in both analyzers^10^.

As is consistent with previous results, in our study, the Galilei G4 Dual and Cassini showed high reproducibility in all corneal power measurements (simK, total, and posterior) in both normal and post-refractive eyes^10,17–22^. For instance, Savini et al. found the ICCs of simK, total, and posterior corneal power to be 0.999, 0.999, and 0.994, respectively, in a group of both normal and post-refractive eyes. Kim et al. recorded similar measurements in normal (0.998, 0.997, 0.992) and post-refractive surgery (0.998, 0.998, 0.990) eyes. This is similar to our values seen using the Galilei in normal (0.998, 0.996, 0.981) and post-refractive surgery (0.997, 0.997, 0.999) eyes and using the Cassini in normal (0.988, 0.989, 0.936) and post-refractive surgery (0.982, 0.988, 0.966) eyes.

In addition, in both normal and post-refractive eyes, repeatability was high for simK and total corneal astigmatism measurements but was low for posterior corneal astigmatism measurements with the Galilei and Cassini. The ICC for posterior corneal astigmatism magnitude measurements in Galilei were 0.616 in normal eyes and 0.669 in post-refractive eyes and with the Cassini 0.554 in normal eyes and 0.380 in post-refractive eyes. This was comparable to previously reported posterior corneal astigmatism magnitudes for both normal eyes 0.499 and post-refractive 0.183 under dual rotating Scheimpflug–Placido and color-LED corneal topography in healthy volunteers with median age of 32 ± 7 years and 32 ± 8 years respectively for normal and post-refractive groups^10^.

These results are in agreement with previous studies that noted either low or moderate repeatability of posterior astigmatism measurements using both the Scheimpflug and Cassini analyzer^9–11,19,20^. The low repeatability may be explained by the low magnitude of posterior corneal astigmatism (Galilei mean − 0.31 ± 0.12 D and − 0.31 ± 0.16 D for normal and post-refractive eyes, respectively; Cassini mean − 0.34 ± 0.17 D and − 0.34 ± 0.17 D for normal and post-refractive eyes, respectively) as described by Kobashi^23^. In addition, the 95% LOA for the Cassini vs Galilei posterior corneal astigmatism were between − 0.38 and 0.3 in normal eyes and − 0.44 to 0.32 in post-refractive eyes. Since the upper limit of the 95% LOA for the Cassini vs Galilei was − 0.38 and -0.44 in normal and post-refractive eyes, respectively; there is only a 5% probability that both analyzers would overestimate the posterior corneal astigmatism measurement by more than -0.38 and − 0.44 D, respectively. Subtle differences in steep and flat meridian locations are difficult to identify in eyes with low astigmatism as compared to eyes with high astigmatism, contributing to the low repeatability.

For the Galilei the posterior keratometric parameter is derived in the 0.5–2.0 mm zone where as for the Cassini posterior corneal measurement is calculated analyzing reflections of seven white LEDs in approximately the 3.0 mm annular region. This difference may account for part of the statistically significant difference (p < 0.01) comparing the mean measurements between the Cassini and Galilei G4 devices for total corneal power in normal eyes and for posterior corneal power in post-refractive eyes. As evidenced in the results of this study, the differences in both devices with respect to anterior astigmatism values in normal and post-refractive eyes were not significant, indicating comparable results between devices. However, with respect to total corneal power in normal eyes and posterior corneal power in post-refractive eyes, the machines were not interchangeable.

Most studies evaluating corneal topography repeatability enrolled only young, healthy patients, primarily between 18 and 40 years of age^9,10,17–19,21,24^. Our study enrolled patients with a mean age of 55 ± 18.5 years, and an age range of 21.5 and 91.5 years. The high repeatability of both the Galilei G4 Dual and Cassini analyzer measurements were comparable to prior studies of young volunteers, re-confirming good performance of both analyzers across a broad clinical setting.

Limitations of our study include: (1) we did not evaluate inter-observer and intersession repeatability, (2) patients with other corneal diseases were excluded and (3) we did not evaluate the repeatability of pachymetry or corneal wavefront aberrations.

In summary, our results indicate that the Galilei G4 Dual and Cassini analyzer both have high repeatability in recording simK, total, and posterior corneal power but low repeatability in posterior corneal astigmatism measurements in patients with and without a history of refractive surgery in the older cataract population.

## Data Availability

The datasets generated during and/or analyzed during the current study are available from the corresponding author on reasonable request.

## Code availability

Statistical analysis was performed using R software (version 0.99.903, R Foundation for Statistical Computing, Vienna, Austria).
